# The relation between sense of coherence and daily hassles among university students

**DOI:** 10.1080/21642850.2018.1538802

**Published:** 2018-10-29

**Authors:** Jacek Hochwälder, Vanja Saied

**Affiliations:** Department of Psychology, Mälardalens University, Eskilstuna, Sweden

**Keywords:** Salutogenic model, sense of coherence, daily hassles, university students, gender differences

## Abstract

Based on Antonovsky’s salutogenic model, it was hypothesized that persons with a high sense of coherence (SOC), compared to persons with a low SOC, (1) experience fewer hassles and (2) experience hassles as less stressful. In addition to each of the two main hypotheses, gender differences and interaction between SOC and gender were also explored. Two hundred and fifty-eight female students (M_age_ = 23.77 years) and 136 male students (M_age_ = 24.02 years) participated in a survey where they responded to a questionnaire that was designed to measure some demographic variables, SOC and daily hassles. The data were analysed by two 3 (SOC-groups) × 2 (Gender) ANOVAs for independent measures, with frequency and intensity as dependent variables, followed up with Tukey’s HSD *post-hoc* tests. The results confirmed both main hypotheses. In addition, the results showed no interaction between SOC and gender, no differences between female and male students with regard to the number of experienced hassles but that female students experienced the hassles more intensively. These findings further corroborated the two fundamental parts of Antonovsky’s salutogenic model concerned with avoidance and appraisal of stressors.

## Introduction

In 1970 Antonovsky was analysing a large dataset from a study on how Israeli women adapt to the menopause, when by chance he noticed that 51% of the women who had not experienced the Nazi-concentration camps had rather good health compared to 29% of the women who had survived the camps. This observation caused him to reflect on what makes some people stay healthy despite having experienced horrible events, and subsequently led him to formulate the *salutogenic model* [from: *salus* (health) and *genesis* (origins)] which focuses on factors that promote health (Antonovsky, 1979, 1987).

In this model, several factors or generalized resistance resources (GRRs) are suggested. Antonovsky (1979, p. 103) defined a GRR as a “physical, biochemical, artifactual-material, cognitive, emotional, valuative-attitudinal, interpersonal-relational, macrosociocultural characteristic of an individual, primary group, subculture, society that is effective in avoiding, combating a wide variety of stressors and thus preventing tension from being transformed into stress.” Furthermore, he also assumed that what they all have in common is that they relate to a sense of consistency, possibility to affect the underload-overload and of being able to shape outcomes in life-experiences (Antonovsky, 1987). The repeated use of the available GRRs to various life-experiences are said to result in a rather stable dispositional orientation termed *sense of coherence* (SOC) (Antonovsky, 1979, 1987).

SOC is the core concept in the model and it is defined by Antonovsky (1987, p. 19) as: “a global orientation that expresses the extent to which one has a pervasive, enduring though dynamic feeling of confidence that: (1) the stimuli, deriving from one’s internal and external environments in the course of living are structured, predictable and explicable; (2) the resources are available to one to meet the demands posed by these stimuli; and (3) these demands are challenges, worthy of investment and engagement”. Thus, a higher SOC indicates that life is being perceived as more: (1) comprehensive (the cognitive component); (2) manageable (the instrumental/behavioral component); and (3) meaningful (the motivational component). It is clearly stated by Antonovsky (1987, 1993) that the degree of SOC is dependent on all three components, and therefore the three components should not be measured separately. Literature reviews shows that SOC tend to increase with age over the life span and that women tend to have little lower SOC than men (Eriksson & Lindström, 2005).

In the model, SOC is assumed to be related to how well people manage to cope with stressors, where stressors are defined as “demands that tax or exceed the resources of the system” (Lazarus & Cohen, 1977, p. 109). More specifically, it is postulated that: (a) a person with a high SOC should be able to *avoid* stressors better than a person with a low SOC; (b) a person with a high SOC should *appraise* stressors as less stressful than a person with a low SOC, and; (c) a person with a high SOC should be able to *manage* to cope more successfully with a stressor than a person with a low SOC (Antonovsky, 1979).

There are various types of stressors. According to Antonovsky, a rough distinction can be made between the following three significant types: (a) *chronical stressors*, a life situation or state/trait that in a relatively permanent and continuous way affects life (e.g. disability); (b) *major life-events*, which can be demarcated in space and time and be characterized primarily not by their occurrence but their consequences (e.g. husband’s or wife’s death); (c) *daily hassles*, rather minor but potentially irritating incidents occurring in everyday life (e.g. an argument with a co-worker) (Antonovsky, 1987).

Antonovsky (1987) stated that he was little interested in daily hassles because he doubted that they had any significant effect on health. However, empirical research has shown that it is not the major life-events but the daily hassles that have the most profound effects on both mental and somatic health (e.g. Burks & Martin, 1983; DeLongis, Coyne, Dakof, Folkman, & Lazarus, 1982; Eckenrode, 1984; Jandorf, Deblinger, Neale, & Stone, 1986; Kanner, Coyne, Schaefer, & Lazarus, 1981; Monroe, 1983; Ruffin, 1993; Weinberger, Hiner, & Tierney, 1987; Zarski, 1984).

The focus of the present study is on how SOC relates to the avoidance and appraisal of daily hassles. These relations have not received any attention in previous research. However, the relation between SOC and avoidance of major life-events has been studied and has shown that a high SOC is associated with the experience of few negative life-events (e.g. Anson, Carmel, Levenson, Bonneh, & Maoz, 1993; Feldt, Leskinen, & Kinnunen, 2005; Jorgensen, Frankowski, & Carey, 1999; Richardson & Ratner, 2005; Surtees, Wainwright, & Khaw, 2006; Volanen, Suominen, Lahelma, Koskenvuo, & Silventoinen, 2007). Furthermore, the relationship between SOC and appraisal of life-events has been investigated only indirectly, and while some studies have shown that a high SOC protects (buffers) against stress when actually experiencing negative life-events (e.g. Bishop, 1993; Jorgensen et al., 1999; Richardson & Ratner, 2005), other studies have not been able to show this protective (buffering) effect (e.g. Carmel, Anson, Levenson, Bonneh, & Maoz, 1991; Flannery & Flannery, 1990).

Much research in behavioral sciences is conducted on university students because it is a convenient way to collect data. However, university students are also of specific interest for this particular study because they are exposed to various types of hassle. This group of young adults are in a decisive and dynamic stage of life that can be characterized by many different demands and transitions with regard to both academic and private life (e.g. Bouteyre, Maurel, & Bernoud, 2007; Nguyen-Michel, Unger, Hamilton, & Spruijt-Metz, 2006). As mentioned above, daily hassles are shown to be related to health, so how well students manage to deal with various daily hassles, besides affecting their health, could also affect their future professional and personal life (e.g. Blankstein & Flett, 1992; Bouteyre et al., 2007). It is therefore important to further investigate the relationship between SOC and hassles amongst female and male university students.

The findings from studies on gender differences with regard to the experience of daily hassles are very difficult to summarize because: (a) often no distinction is made between daily hassles and major life-events; (b) different collections of hassles are used; (c) different types of measures are calculated based on all, different types, or specific hassles or life-events; (d) different samples of participants are studied (e.g. of various ages). However, research on adolescence has shown that girls, compared to boys, experience more hassles (e.g. Bobo, Gilchrist, Elmer, Snow, & Schinke, 1986; Kohn & Milrose, 1993; Lai, Hamid, & Chow, 1996; Wagner & Compas, 1990) and experience them as more stressful (e.g. Kanner, Feldman, Weinberger, & Ford, 1987; Lai et al., 1996; Wagner & Compas, 1990; Wu & Lam, 1993). For university students, these relations have not been thoroughly investigated and have given mixed results: in one study, no gender differences were found either with regard to the number of experienced hassles or how intensively the encountered hassles were experienced (Sarafino & Ewing, 1999). In another study, it was found that female students experienced hassles more intensively than male students (Blankstein & Flett, 1992). Based on the more extensive and rather univocal findings for adolescence, it is suggested that female students, as compared to male students, should be expected to report more hassles and experience them as more stressful. Furthermore, the findings for gender differences in combination with the findings that low SOC is related to experiencing more negative life-events and often experiencing the negative life-events as more stressful would suggest that being a female student *and* having a low SOC may result in extra vulnerability with regard to number of experienced hassles and how intensively the encountered hassles will be experienced (cf. Eriksson, 2015; Myrin & Lagerström, 2008).

The aim of the present study was to test the following two main hypotheses that were derived from Antonovsky’s salutogenic model (Antonovsky, 1979, see especially pp. 184–185).

1. *The avoidance (frequency) hypothesis*: Students with a high SOC experience fewer daily hassles (defined as the total number of different hassles experienced during the past month), compared to students with a low SOC. This hypothesis tested one of the postulates in the salutogenic model that states that persons with a high SOC avoid stressors to a greater extent than persons with a lower SOC (Antonovsky, 1979, see pp. 184–185).
2. *The appraisal (intensity) hypothesis*: Students with a high SOC experience the daily hassles as less stressful (defined as the average intensity of the hassles that were actually encountered during the past month), compared to students with a low SOC. This hypothesis also directly tested one of the postulates in the salutogenic model that states that persons with a high SOC define stimuli as non-stressors to a greater extent compared to persons with a lower SOC (Antonovsky, 1979, see pp. 184–185).

In addition to each of the two above stated main hypotheses, the following two tentative hypothesis concerned with gender differences and interactions between gender and SOC were explored.

1. Female students, as compared to male students, are expected to report more hassles and experience them as more stressful.
2. Female students having a low SOC, as compared to groups having other combinations between gender and the three SOC-level, are expected to report more hassles and experience them as more stressful.

## Method

### Participants

Four hundred and nine students at a university in Sweden were asked to participate in this study, of which 394 agreed. One hundred and twenty (30.5%) studied psychology, 82 (20.8%) economy, 47 (11.9%) pedagogy, 38 (9.6%) civil engineering, 37 (9.4%) physiotherapy, 37 (9.4%) nursing, and 33 (8.45%) behavioral sciences. Two hundred and fifty-eight were women (65.5%) with an average age of 23.77 years (SD = 5.18 years) and 136 were men (34.5%) with an average age of 24.02 years (SD = 4.26 years).

### Questionnaire

After having indicated their gender, age, and the subject currently studied, the participants were asked to give their responses with regard to SOC and daily hassles.

#### Sense of coherence

SOC was measured by a scale developed by Antonovsky (1987, 1993). The scale consists of 29 items (e.g. “How often do you have the feeling that there is little meaning in the things you do in your daily life?”) and responses are given on a 7-point (1–7) scale. There are several reasons that an overall value on this scale should be computed. (a) A theoretical reason given by Antonovsky (1987, 1993) is that the degree of SOC is dependent on all three components, and therefore the three components should not be measured separately. (b) A methodological reason is that the use of facet-design (cf., Guttman, 1974; Shye, 1978) in the development of the scale makes it inappropriate to separate the three components. (c) Finally, the psychometric properties of the SOC-scale have been systematically reviewed and it was concluded that the scale is reliable and valid but that the factorial structure of the scale is not clear (Antonovsky, 1993; Eriksson & Lindström, 2005) though some studies have showed that an overall value should be computed (e.g. Klepp, Mastekaasa, Sorensen, Sandanger, & Kleiner, 2007). Thus, for each participant, a total score was computed by adding up the scores for the 29 items, where higher values indicated a higher degree of SOC (with a theoretical range of 29 to 203). The Cronbach’s alpha coefficient for the SOC-scale in this study was 0.89.

Both conceptually (Antonovsky, 1987) and operationally (e.g. Anson et al., 1993; Karlsson, Berglin, & Larsson, 2000; Ristakari, Sourander, Ronning, Nikolakaros, & Helenius, 2008; Surtees et al., 2006) participants are often categorized into groups with low, moderate and high SOC. The reason for this trichotomization is to be able to compare groups that are at the extreme ends of SOC (that is, persons with a high SOC compared to those with a low SOC). In the present study, the participants were divided into three approximately equally large groups based on the 33rd and the 66th percentiles. Participants with a SOC below the 33rd percentile (SOC ≤ 126) were categorized as having a low SOC, participants with a SOC between the 33rd and 66th percentile (127 ≤ SOC ≤ 144) were categorized as having a moderate SOC, and those with a SOC above the 66th percentile (SOC ≥ 145) were categorized as having a high SOC.

#### Daily hassles

The retrospective experience of daily hassles was measured by a scale developed by Maybery (2003). The scale aims to capture the possible daily hassles encountered specifically by university students. The scale consists of 64 hassles that fall into 15 domains related to problems with: friends (4 items), spouse/partner/boy/girlfriend (8 items), children (4 items), lecturers (4 items), parents (4 items), students (4 items), relatives (4 items), employer (4 items), work (5 items), money (4 items), study time pressures (4 items), health (5 items), academic limitations (3 items), course interest (2 items), and finding a job (5 items). For each of the 64 items, the participants were asked to select one of the six responses, with reference to the past month: “had not occurred” (=0), “occurred, not severe” (=1), “occurred, somewhat severe” (=2), “occurred, moderately severe” (=3), “occurred, very severe” (=4), “occurred, extremely severe” (=5). The preliminary findings, concerning the scale’s psychometric properties, have shown that it is valid, highly reliable and has a clear factorial structure (Maybery, 2003). The Cronbach’s alpha coefficient for the hassle instrument in this study was 0.94.

As in some previous studies (e.g. Kanner et al., 1981; Sarafino & Ewing, 1999), two scores were computed for each participant based on his/her responses to the 64 items. (1) Frequency-score: was constructed to reflect the number of different hassles experienced during the past month. The score was simply computed as the number of different hassles encountered (theoretical range of 0 to 64). (2) Intensity-score: was constructed to reflect how intense, on average, the participant experienced a hassle that was encountered during the past month. The score was computed by taking the sum of the severity rating for the encountered hassles, divided by the number of encountered hassles (theoretical range of 1 to 5).

### Procedure

The participants were selected using convenience sampling. Lecturers at various departments were asked if data collection could be carried out in connection with some of their lectures. Before the lectures, the students were briefly informed about the purpose of this study, that no compensation would be given for their participation, and also that their participation was anonymous and voluntary. The questionnaire usually took 15–20 min to complete.

### Analyses

All the descriptions and analyses were performed using the SPSS program (e.g. SPSS, 1990). Descriptive statistics in form of arithmetic means, standard deviations, Pearson’s correlation coefficients and Cronbach’s alpha coefficients were calculated. To test the hypotheses, the data were analysed using two separate 3 (SOC-groups) × 2 (Gender) ANOVAs for independent measures, with frequency and intensity as the dependent variables, followed up with Tukey’s HSD *post-hoc* tests. (Because SOC tends to increase over the entire life span (e.g. Eriksson & Lindström, 2005), initially covariance analyses with age as covariate were performed. However, because these analyses gave almost the same results as the ANOVAs, the results from the ANOVAs are presented.).

## Results

### Descriptive statistics

Table 1 shows arithmetic means, standard deviations, and Pearson’s correlation coefficients for variables in this study. As can be seen, there was a rather weak negative linear correlation between SOC and the two types of ratings of the hassles. The correlation between frequency and intensity was positive but rather low.

**Table 1.** Descriptive statistics for the variables.

Variable	*M*	*SD*	*r*				
			1	2	3	4	5
1. Gender^a^	n.a.^b^	n.a.^b^	–				
2. Age	23.86	4.88	−.02	–			
3. SOC	134.58	21.41	−.02	.00	–		
4. Frequency	23.99	12.63	.01	.14**	−.31***	–	
5. Intensity	2.45	0.67	.14**	−.02	−.37***	.25***	–

Note: *n* = 394; *p < .05; **p < .01; ***p < .001; ^a^men = 0, women = 1; ^b^n.a. = not applicable.

### Frequency ratings

Table 2 presents means and standard deviations for frequency ratings. As could be previously seen in Table 1, those with higher SOC experienced daily hassles significantly less frequently (*r* = −.31, *p* < .001). Now examining the differences between the three SOC-groups, the ANOVA with frequency ratings as the dependent variable indicated significant differences between the three SOC-groups, *F*_2,388 _= 14.55, *p* < .001, *η*^2^ = .07. Tukey’s HSD *post-hoc* tests indicated significant differences between the low and the moderate SOC-group (*p* < .01), between the low and the high SOC-group (*p* < .001) and between the moderate and the high SOC-group (*p* < .01), which means that the high SOC-group experienced least hassles, followed by the moderate SOC-group, that in turn was followed by the low SOC-group (see Table 2). There were no significant differences between men and women with regard to frequency ratings, *F*_1,388 _= .04, *p* = .83. Finally, there was no significant interaction between SOC and gender on the frequency ratings, *F*_2,388_ = .19, *p* = .82.

**Table 2.** Means and standard deviations for frequency.

SOC	Men			Women			Total		
	*M*	*SD*	*n*	*M*	*SD*	*n*	*M*	*SD*	*n*
Low	27.52	14.65	46	28.89	10.48	80	28.39	12.13	126
Moderate	24.11	13.32	44	24.10	13.48	96	24.11	13.38	140
High	19.89	10.85	46	19.34	10.66	82	19.54	10.69	128
Total	23.84	13.32	136	24.07	12.29	258	23.99	12.64	394

### Intensity ratings

Table 3 presents means and standard deviations for intensity ratings. As could be previously seen in Table 1, those with higher SOC experienced daily hassles significantly less intensively (*r* = −.37, *p* < .001). Now examining the differences between the three SOC-groups, the ANOVA with intensity ratings as the dependent variable indicated significant differences between the three SOC-groups, *F*_2,388_ = 18.05, *p* < .001, *η*^2 ^= .08. Tukey’s HSD *post-hoc* tests indicated significant differences between the low and the moderate SOC-group (*p* < .001), between the low and the high SOC-group (*p* < .001) and between the moderate and the high SOC-group (*p* < .05), which means that the high SOC-group experienced, on average, the hassles least intensively, followed by the moderate SOC-group, that in turn was followed by the low SOC-group (see Table 3). On average, women experienced the hassles more intensively than men, *F*_1,388_ = 9.99, *p* < .001, *η*^2 ^= .025. Finally, there was no significant interaction between SOC and gender on the intensity ratings, *F*_2,388 _= 2.65, *p* = .07.

**Table 3.** Means and standard deviations for intensity.

SOC	Men			Women			Total		
	*M*	*SD*	*n*	*M*	*SD*	*n*	*M*	*SD*	*n*
Low	2.47	0.67	46	2.88	0.65	80	2.73	0.68	126
Moderate	2.39	0.70	44	2.42	0.61	96	2.41	0.64	140
High	2.09	0.54	46	2.28	0.61	82	2.21	0.59	128
Total	2.32	0.65	136	2.51	0.67	258	2.45	0.67	394

## Discussion

Both main hypotheses, which were derived from the salutogenic model (Antonovsky, 1979), were supported. Students with a high SOC, compared to students with a lower SOC, experienced: (1) fewer daily hassles (the avoidance (frequency) hypothesis); (2) daily hassles as less stressful (the appraisal (intensity) hypothesis). Furthermore, female and male students did not differ with regard to the number of experienced hassles but female students experienced the hassles more intensively. There were no significant interactions between SOC and gender on the frequency ratings and intensity ratings.

These findings give further support to the postulates that persons with a high SOC, compared with persons with a lower SOC, more easily can avoid stressors and appraise stimuli as less stressful (Antonovsky, 1979). While the obtained results regarding the relation between SOC and avoidance of hassles are in line with previous findings on the relation between SOC and life-events (e.g. Anson et al., 1993; Feldt et al., 2005; Jorgensen et al., 1999; Richardson & Ratner, 2005; Surtees et al., 2006; Volanen et al., 2007), the second hypothesis have not been tested previously. One practical implication of these obtained results is that in working with stress related health issues among students, the SOC-scale could be used to identify the more vulnerable students (those with low SOC).

In addition, no significant interaction between SOC and gender were noted and also no gender differences were noted with regard to the number of experienced hassles, a result in line with Sarafino and Ewing’s (1999) findings. Female students, compared to male students, experienced the hassles more intensively, a result in line with Blankstein and Flett’s findings (1992) but contrary to those of Sarafino and Ewing (1999). However, it is not necessarily the case that female students actually experienced the hassles more intensively. An alternative interpretation is that they were more ready to express vulnerable feelings or in other words, that it is more socially acceptable for women to express such feelings (e.g. Lai et al., 1996). One implication of the gender differences found in the present study is that female students may generally be more vulnerable than male students, when exposed to daily hassles.

The present study has some strengths. Firstly, it should be noted that the aim of this study was to test the relation between SOC and the two aspects of *hassles in general* (that is, without dividing hassles into different categories) and not how SOC is related to the two aspects *in various types of hassle* (that is, when hassles are divided into different categories). The frequency and intensity were therefore computed over a large sample of hassles from different domains, which should contribute to a valid estimate of the general relation between SOC and the three aspects of hassles. Secondly, previous research has stressed the importance of using hassles relevant to the persons studied (e.g. Blankstein & Flett, 1992; Blankstein, Flett, & Koledin, 1991). The hassles used in the present study were compiled specifically for university students, which should contribute to the ecological validity of this study.

The present study also has some limitations. Firstly, Antonovsky stated that SOC is a rather stable dispositional orientation (Antonovsky, 1987) but empirical studies have only given moderately strong support for this statement (e.g. Eriksson & Lindström, 2005). Even though it is assumed that it is SOC that affects the two aspects of daily hassles, in the present cross-sectional study only statistical, and not causal, relations between SOC and the two aspects of hassles have been proven. Secondly, the participants were divided into three groups of SOC based on percentiles, which is a method often used (e.g. Anson et al., 1993; Karlsson et al., 2000; Magnusson, 1967; Ristakari et al., 2008; Surtees et al., 2006) mainly because there are no well-established cut-off points for SOC (e.g. Eriksson & Lindström, 2005). The shortcoming with this approach is that the cut-off points vary between studies and also that the differentiation between the three groups will vary. Thirdly, as in previous studies on daily hassles (e.g. Kanner et al., 1981; Sarafino & Ewing, 1999), daily hassles were measured retrospectively. These retrospectively rated experiences can, of course, differ from the actual experiences of daily hassles. Fourthly, a relatively large sample of both female and male students from different departments participated in the present study making the results likely to be generalizable to university students. However, the obtained results for this particular convenience sample of university students cannot automatically be generalized to other populations that may experience different types of hassles.

In order to build on the results of the present study – besides dealing with the above-mentioned limitations – future research should focus on the following issues. Firstly, study how SOC and gender (separately and in combination) are related to the frequency and intensity of specific types of hassle as well as to uplifts, defined as positive experiences in daily life, such as hearing good news or receiving a compliment (e.g. Kanner et al., 1981). Secondly, make a clear distinction between hassles that are inevitable and beyond one’s control to influence (e.g. deadline on a course assignment) and the ones that are possible to avoid and control (e.g. conflict with another student).

The practical implication of the present findings is that an effort should be made to promote a higher SOC in students in order to make the impact of daily hassles less harmful. Antonovsky (1987) postulated that SOC becomes a relatively permanent disposition first around 30 years of age, but unfortunately (to our knowledge) there are no programs showing how to promote SOC in children and adolescents. However, based on the salutogenic model as a guide to health promotion (Antonovsky, 1996) the aim should be to supply the growing individual with GRRs and to try to make the interaction with the environment more harmonious with regard to consistency or predictability (enhancing comprehensiveness), underload-overload balance (enhancing manageability) and participation in shaping outcomes (enhancing meaningfulness). For some guidance on how such intervention program could be designed, see for example, Langeland (2007).

To conclude, the present findings further corroborate fundamental parts of Antonovsky’s salutogenic model (Antonovsky, 1979) with regard to the relation between SOC and avoidance and appraisal of stressors in the form of daily hassles.
